# 60 A Maladaptive Fibrinolytic Phenotype Is Associated with Inflammatory Cytokines and Mortality After Burn Injury

**DOI:** 10.1093/jbcr/iraf019.060

**Published:** 2025-04-01

**Authors:** Amanda Soo Ping Chow, Tuan Le, Anthony Pusateri, Desiree Pinto, Melissa McLawhorn, Lauren Moffatt, Jeffrey Shupp

**Affiliations:** The Burn Center at MedStar Washington Hospital Center; MedStar Health Research Institute; Naval Medical Research Unit San Antonio; The Burn Center at MedStar Washington Hospital Center; MedStar Health Research Institute; MedStar Health Research Institute; MedStar Washington Hospital Center

## Abstract

**Introduction:**

Burn injury results in a multifactorial response characterized by dysregulation of the inflammatory and fibrinolytic systems. This inflammatory response is characterized by changes in the levels of cytokines with elevated levels of pro-inflammatory cytokines portending a poorer prognosis. Abnormal fibrinolytic function following burn injury is also associated with mortality risk. However, the relationship between fibrinolytic function and the inflammatory response up to 48 hours post-burn has not been established. This study aimed to identify a relationship between a maladaptive fibrinolytic phenotype and inflammatory cytokines, and their association with mortality.

**Methods:**

Patients presenting to a regional burn center within 4 hours of thermal injury were included. Blood was drawn at hospital admission and sequentially at hours 2, 4, 8, 12, 24, 36, and 48. Thromboelastography (Rapid TEG) was performed. Percent clot lysis 30 minutes after maximum clot strength (LY30) was used to characterize fibrinolytic phenotypes: shutdown (SD; < 0.6), physiologic (PHYS; 0.6-7.7), and hyperfibrinolytic (HF; >7.7). Phenotype patterns that remained in or transitioned to HF or SD at hour 48, or the latest available time point were considered maladaptive. A multivex assay was used to obtain levels of interleukin 6 (IL-6), interleukin 10 (IL-10), and tumor necrosis factor alpha (TNF-α). Cytokine patterns by fibrinolytic phenotypes and mortality status were examined using mixed effects model.

**Results:**

Of 98 patients included, the mean TBSA burned was 20.9% (21.8). Overall mortality was 14.3%. From admission to hour 48, levels of IL-6 and TNF-α increased from 73.1 to 178.9 pg/mL and 1.3 to 1.8 pg/mL respectively, while levels of IL-10 decreased from 23.5 to 7.5 pg/mL, but there were no statistically significant differences in cytokine levels among the three fibrinolytic phenotypes at any time point. A maladaptive phenotype pattern (n=34, 34.7%) was associated with higher mortality compared to those with an adaptive phenotype (n=64, 65.3%) (26.5% vs 7.8%; p=0.02). Compared to an adaptive phenotype, IL-6 levels were higher at hours 24 (443.1 vs 121.8 pg/mL; p=0.03) and 36 (436.1 vs 90.2 pg/mL; p=0.02), while IL-10 levels were higher at hours 8 (7.2 vs 1.5 pg/mL; p=0.03) in patients with a maladaptive phenotype. Levels of TNF-α were not different between phenotypes.

**Conclusions:**

After burn injury, a maladaptive fibrinolytic phenotype is associated with elevated IL-6 and IL-10, and mortality. Fibrinolytic dysregulation is associated with increased cytokine response in severe burn. Further biochemical studies are needed to explore the relationships between fibrinolysis and inflammation.

**Applicability of Research to Practice:**

Correction or treatment of burn induced fibrinolytic dysfunction may mitigate the hyperinflammatory response after burn injury.

**Funding for the Study:**

Department of Defense Award Number W911NF-10-1-0459

